# Effect of *Byrsonima crassa* and Phenolic Constituents on *Helicobacter pylori-*Induced Neutrophils Oxidative Burst

**DOI:** 10.3390/ijms13010133

**Published:** 2011-12-23

**Authors:** Cibele Bonacorsi, Maria Stella G. Raddi, Luiz Marcos da Fonseca, Miriam Sannomiya, Wagner Vilegas

**Affiliations:** 1 School of Pharmaceutical Sciences, São Paulo State University (UNESP), Araraquara, SP, 14801-902, Brazil; E-Mail: bonacorsic@yahoo.com.br; 2 School of Arts, Sciences and Humanities, University of São Paulo (USP), São Paulo, SP, 03828-000, Brazil; E-Mail: miriamsannomiya@yahoo.com.br; 3 Institute of Chemistry, São Paulo State University (UNESP), Araraquara, SP, 14800-900, Brazil; E-Mail: vilegasw@gmail.com

**Keywords:** *Byrsonima crassa*, *Helicobacter pylori*, gastric ulcers, antioxidant activity, phenolic compounds, chemiluminescence

## Abstract

*Byrsonima crassa* Niedenzu (Malpighiaceae) is used in Brazilian folk medicine for the treatment of diseases related mainly to gastric ulcers. In a previous study, our group described the gastric protective effect of the methanolic extract from the leaves of *B. crassa*. The present study was carried out to investigate the effects of methanolic extract and its phenolic compounds on the respiratory burst of neutrophils stimulated by *H. pylori* using a luminol-based chemiluminescence assay as well as their anti-*H. pylori* activity. The suppressive activity on oxidative burst of *H. pylori*-stimulated neutrophils was in the order of methyl gallate > (+)-catechin > methanol extract > quercetin 3-*O*-α-l-arabinopyranoside > quercetin 3-*O*-β-d-galactopyranoside > amentoflavone. Methyl gallate, compound that induced the highest suppressive activity with IC_50_ value of 3.4 μg/mL, did not show anti-*H. pylori* activity. *B. crassa* could be considered as a potential source of natural antioxidant in gastric ulcers by attenuating the effects on the damage to gastric mucosa caused by neutrophil generated reactive oxygen species, even when *H. pylori* displays its evasion mechanisms.

## 1. Introduction

The discovery of *Helicobacter pylori* (*H. pylori*) in 1982 was the starting point of a conceptual revolution concerning of gastroduodenal diseases and their management [1]. *H. pylori* infection has been implicated in the pathogenesis of chronic gastritis, peptic ulcers and, more rarely, gastric cancer and gastric lymphoma of mucosa-associated lymphoid tissue [2]. The mechanisms by which bacterial infection leads to gastric mucosal damage include the direct effects of virulent factors produced by *H. pylori*, the propagation and perpetuation of inflammation, oxidative stress, and the induction of apoptosis in infected gastric epithelial cells [3].

Under inflammatory conditions, phagocytosing cells generate multiple well-defined reactive oxygen species (ROS). Stimulated polymorphonuclear neutrophils (PMNs) undergo an oxidative burst, and release large quantities of superoxide anion as a result of the activation of the PMN NADPH oxidase. Superoxide anion radicals are known to dismutate to form hydrogen peroxide (H_2_O_2_) and oxygen [4]. Myeloperoxidase (MPO), an enzyme released from the azurophilic granules in neutrophils, uses H_2_O_2_ and chloride ions (Cl^−^) as substrates to produce hypochlorous acid (HOCl), an important antibacterial compound, but an extremely strong oxidant that can also attacks host biomolecules [5]. Oxidative stress plays a critical role in the augmented mucosal damage caused by *H. pylori* infection, and an antioxidant could ameliorate the aggravation caused by stress-associated gastric mucosal damage.

Despite years of experience with *H. pylori* treatment, an ideal regimen to treat the infection has not yet been identified. The most effective treatment is a combination of a proton pump inhibitor and antibiotics, but this fails to eradicate the infection in 10–20% of patients [6]. Non-antibiotic treatments, including phytomedicines, probiotics and antioxidants, have been increasingly investigated as potential alternatives for the treatment of *H. pylori* infection [7,8].

*Byrsonima crassa* (Malpighiaceae) is a plant found in the Cerrado of the central region of Brazil and is used in folk medicine for the treatment of gastroduodenal diseases, including gastric ulcers. Pharmacological studies have revealed that *Byrsonima* species have an antiulcerogenic effect and that the methanolic extract of leaves from *B. crassa* has a gastroprotective effect against HCl/ethanol-induced gastric mucosal injuries in mice. Amentoflavone, catechin and quercetin derivatives have been suggested to be the active components of the extract [9].

Considering that *B. crassa* is commonly used as a phytomedicine to treat ulcers and gastritis, the objective of the present study was to evaluate the effect of the extract and its purified major phenolic constituents (amentoflavone, (+)-catechin, methyl gallate, quercetin 3-*O*-α-l-arabinopyranoside, and quercetin 3-*O*-β-d-galactopyranoside) on the oxidative burst of PMNs stimulated by *H. pylori*, using a luminol-dependent chemiluminescence assay, and the anti-*H. pylori* activity of each compound.

## 2. Results and Discussion

There is considerable interest in alternative approaches to the treatment of *H. pylori*, such as the use of biologically active compounds, including antimicrobials compounds and antioxidants from plants. In a previous study, the anti-ulcerogenic activity profile of *B. crassa* leaves was investigated in detail, in various *in vivo* experimental ulcer models [9]. In this study the effects of the whole extract and purified phenolic compounds on the intra- and extracellular production of ROS was assessed in *H. pylori-*stimulated PMNs using luminol-enhanced chemiluminescence. Luminol can be oxidized by several ROS, but it is generally accepted that chemiluminescence in neutrophils results from intra- and extracellular events and depends mainly on the reactions of the MPO-H_2_O_2_-Cl system [10]. The suppressive activity on oxidative burst of *H. pylori*-stimulated PMNs was in the order of methyl gallate > (+)-catechin > methanol extract > quercetin 3-*O*-α-l-arabinopyranoside > quercetin 3-*O*-β-d-galactopyranoside > amentoflavone. All compounds showed a dose-dependent effect (Table 1).

In general, the free-radical scavenging and antioxidant activities of phenolics depend primarily on the number and positions of the hydrogen-donating hydroxyl groups on the aromatic ring of these molecules, but is also affected by other factors, such as glycosylation of aglycones and other H-donating groups [11]. The experimental results of this study showed that the flavonoid quercetin (standard antioxidant) had a better antioxidant activity than its 3-O-glycoside derivatives (quercetin 3-*O*-α-l-arabinopyranoside and quercetin 3-*O*-β-d-galactopyranoside) on ROS production induced by *H. pylori*. These agree with reports that flavonoid aglycones are more potent antioxidants than their corresponding glycosides [12].

Methyl gallate has been shown to be an effective antioxidant in a variety of acellular experiments [13]. In this study, this gallic acid derivative showed a strong inhibitory activity on the induced oxidative stress of PMNs using *H. pylori* as activator. The molecular mechanism for ROS production by *H. pylori* remains unclear. Analysis of intracellular ROS shows that methyl gallate is effective in attenuating H_2_O_2_-derived ROS [14]. The antioxidant-like properties of polyphenols are largely dependent on the type of stimulus for the production of ROS and the structure plays a critical role in the success as an antioxidant. The results of ROS inhibitory activity of different phenolic compounds are indicative of different action mechanisms and further researches are required to understanding their action on ROS induced by *H. pylori* in neutrophils. One of the initial components of the innate immune response to be encountered by *H. pylori* in the stomach is the gastric epithelial cell [3]. As *B. crassa* and phenolic constituents have impact on ROS induced by PMNs is expect that this benefits can also be extent to the gastric mucosal cells.

In this study the anti-*H. pylori* activity of the phenolic compounds isolated from the methanolic extract of *B. crassa* was also examined (Table 2). All purified compounds tested showed lower anti-*H. pylori* activity than did the whole extract as cited by Bonacorsi *et al*. [15]. Studies have demonstrated the anti-*H. pylori* effect of natural compounds [16,17]. These compounds interact with multiple molecular targets and inhibit the growth of *H. pylori* by various mechanisms such, as membrane destabilization, inhibition of ion channels and inhibition of bacterial metabolism [14]. Phenolic compounds isolated from *B. crassa* weakly inhibited *H. pylori* growth. Shin *et al*. [18] have previously reported that some flavonoids, such catechins, quercetin, and naringenin, exhibit poor inhibition on *H. pylori* growth.

Synergistic antimicrobial activity has been demonstrated in some naturally occurring flavonoids. Arima *et al*. [19] reported that the use of combinations of quercetin and quercitrin, quercetin and morin, and quercetin and rutin were more effective against *S. enteritidis* than the use of each flavonoid alone. In a study of the effects of cranberry fruit on *H. pylori*, Vattem *et al*. [20] report the low efficacy of purified phenolics in inhibiting the bacteria compared to the whole fruit extract at a similar dose, suggesting the ability of phenolics to function synergistically in the whole food. The purified phenolic compounds tested showed a lower antimicrobial activity compared to the extract (MIC 1024 μg/mL) [15]. This result might reflect the synergistic interaction of constituent phytochemicals.

## 3. Experimental Section

### 3.1. Plant Material

*B. crassa* leaves were collected at Porto Nacional, TO, Brazil. Authentication was achieved by comparison with a specimen at the herbarium of Tocantins University. A voucher specimen (Nr. 3377) was deposited at the herbarium.

### 3.2. Extract Preparation and Isolation of Purified Phenolic Compounds

The air-dried and powdered leaves (2.0 kg) of *B. crassa* were extracted with methanol (MeOH) at room temperature (48 h). The solvent was evaporated at 60°C under reduced pressure to produce themethanolic extract. The yield (w/w) of the extract from the dried powdered *B. crassa* leaves was 7.91% (158.3 g). An aliquot of the extract (4.0 g) was permeated on a Sephadex LH-20 column (100 cm × 5 cm), and then eluted with MeOH. Fractions (8 mL) were collected and analyzed by thin-layer chromatography on silica gel eluted with CHCl_3_/MeOH (80:20) and revealed by spraying with either (diphenylaminoborate/polyethyleneglycol) or an anisaldehyde/sulfuric acid solution. Fractions 129–141 (95.0 mg) were purified by repeated column chromatography (CC) on microcrystalline cellulose using with CHCl_3_/MeOH (80:20) as the eluent, yielding the biflavonoid amentoflavone (6.0 mg). Fractions 88–95 (69.0 mg) were further purified by high-performance liquid chromatograpy (HPLC), with MeOH/H_2_O (1:1) as the eluent, to yield quercetin-3-*O*-β-d-galactopyranoside (15.0 mg). Fractions 82–87 (122.0 mg) were purified by silica CC using EtOAc/*n*-PrOH/H_2_O 140:8:80 (upper phase) as the eluent, yielding quercetin-3-*O*-α-l-arabinopyranoside (14.0 mg) and a mixture of (−)-epicatechin and (+)-catechin (30.0 mg). Epicatechin and (+)-catechin were separated by HPLC with MeOH/H_2_O (20:80) as the eluent to yield 10 mg of each purified compound. Fractions 55–60 (112.0 mg) were purified by silica gel CC with CHCl_3_/MeOH (75:25) as the eluent, yielding methyl gallate (8.0 mg), which was confirmed by NMR and TLC [21]. The extract and the purified major constituents were solubilized in dimethyl sulfoxide (DMSO).

### 3.3. Anti-Helicobacter pylori Activity

*H. pylori* type strain ATCC 43504, which is metronidazole resistant (MtzR) and amoxicillin susceptible (AmxS), was obtained from the American Type Culture Collection (Manassas, VA, USA). The bacterium was cultured in Columbia agar containing 5% sheep’s blood at 36–37 °C for 3 days under a microaerophilic atmosphere. The antimicrobial activity was determined by a broth microdilution method with brain heart infusion broth supplemented with 10% heat-inactivated fetal bovine serum, as described by Bonacorsi *et al*. [15]. Briefly, the wells of a 96-well microplate were filled with 100 μL of various concentrations of the phenolic compounds (final concentrations of 64 to 1024 μg/mL). Then, an equal volume of *H. pylori* suspension (1 × 10^6^ cfu/mL) was added to each well. The absorbance was determined in an automatic ELISA microplate reader (Spectra & Rainbow Readers, Tecan) at wavelength of 620 nm. The microplate was incubated at 36–37 °C for 3 days under a microaerophilic atmosphere, after which time the plate was shaken and the absorbance was read again, at the same wavelength. Readings obtained before and after incubation were compared, to determine an increase in bacterial growth. Additionally, under the same conditions, wells without test substances were inoculated with *H. pylori*, as positive controls, and uninoculated media were used as negative controls. The percentage of growth inhibition was estimated with respect to a control that was incubated only with the solvent (DMSO). Quercetin (Sigma, USA) was used as a phenolic compound reference. All tests were performed in triplicate and repeated at least three times.

### 3.4. Isolation of Polymorphonuclear Neutrophils

Peritoneal polymorphonuclear neutrophils (PMNs) were obtained from male rats (*Rattus norvegicus albinus*) by intraperitoneal injection of 10 mL of a solution of sterile oyster glycogen 0.5% (w/v) in saline. Twelve hours later, the peritoneal exudate was collected with 20 mL Dulbecco’s phosphate-buffered saline (D-PBS) without calcium containing 10 IU heparin/mL. The cells were washed twice with sterile D-PBS and were carefully layered onto 5 mL of Ficoll-Paque™ (*d* = 1077) and centrifuged at 800 g for 30 min. Subsequently, the PMNs were washed again with D-PBS and adjusted to a concentration of 2.0 × 10^6^ cells/mL. The proportion of neutrophils (over 95%) and cell viability in the peritoneal exudate were determined by cell staining with May-Grünwald-Giemsa. The Ethical Committee of the Pharmaceutical Sciences—UNESP approved the experimental procedure of this study (resol 05/2008).

### 3.5. Luminol Chemiluminescence Assay

The effects of the extract and chemical compounds on the oxidative burst of PMNs were determined by using a luminol-dependent chemiluminescence assay as described by Galice *et al*. [22], with modifications. The chemiluminescence was measured with an automated luminometer (BioOrbit model 1251), using a final reaction volume of 1.0 mL. Briefly, 2.0 × 10^6^ cells/mL and 2.0 × 10^−5^ M luminol were added to tubes containing D-PBS. The stimulus (*H. pylori* suspension at an optical density of 0.2 at 620 nm) was added to the tubes, and light release (in mV) was measured for 15 min. After this, D-PBS containing the extract or the phenolic compounds (non-cytotoxic concentrations) was added, and the oxidative burst was continuously monitored for another 75 min. The chemiluminescence response was quantified as the integrated area below the resulting chemiluminescence curve (AUC), over a period of 0 to 90 min. The background chemiluminescence from PMNs in the absence of stimulus (*H. pylori*) was also measured. Quercetin was used as the antioxidant standard. All tests were performed in triplicate and repeated at least three times. The percentage of chemiluminescence inhibition achieved with each sample was calculated by the formula: [1 – (AUC of the tested sample/AUC of the negative control)] *×* 100. This value was employed in the calculation of IC_50_, which measures the concentration of sample that inhibits 50% of the chemiluminescence produced by PMNs.

### 3.6. Statistical Analysis

The statistical significance of the differences between groups was assessed by analysis of variance (ANOVA), *p*-values < 0.05 were considered significant.

## 4. Conclusions

The present investigation constitutes the first quantitative screening for the effects phenolic compounds on the oxidative burst of PMNs induced by *H. pylori*. It becomes clear that *B. crassa* may exert a protective effect by inhibiting the mechanism by which *H. pylori* and neutrophils collaborate to cause gastric mucosal damage. These results confirm the antioxidant activity of *B. crassa* that justify its use in non-conventional medicine by the Brazilian population for the treatment of gastroduodenal ulcers, particularly when *H. pylori* displays its evasion mechanism.

## Figures and Tables

**Table 1:** Effect of the methanolic extract and phenolic compounds from *Byrsomina crassa* on neutrophil oxidative burst stimulated by *Helicobacter pylori* strain ATCC 43504.

Compound	Concentration (μg/mL)	IA a	% reduction in IA b	IC_50_ (μg/mL)
Methanolic extract	0 (control)	3.15 × 10^5^ ± 22,334	-	27.0
	5	2.19 × 10^5^ ± 20,163	30.5 *	
	50	0.22 × 10^5^ ± 1982	93.0 *	
	100	0.10 × 10^5^ ± 956	96.8 *	

Amentoflavone	0 (control)	2.58 × 10^5^ ± 5482	-	92.9
	1	2.44 × 10^5^ ± 5173	5.4	
	5	2.15 × 10^5^ ± 4355	16.7 *	
	50	1.76 × 10^5^ ± 3760	31.8 *	
	100	1.13 × 10^5^ ± 2407	56.2 *	

(+)-Catechin	0 (control)	2.23 × 10^5^ ± 3363	-	25.8
	1	2.37 × 10^5^ ± 5173	0	
	5	1.6 × 10^5^ ± 2297	28.3 *	
	50	0.10 × 10^5^ ± 146	95.5 *	
	100	0.05 × 10^5^ ± 70	97.8 *	

Methyl gallate	0 (control)	2.75 × 10^5^ ± 7764	-	3.4
	1	2.62 × 10^5^ ± 7404	4.7	
	5	0.74 × 10^5^ ± 2094	73.1 *	
	50	0.15 × 10^5^ ± 423	94.5 *	
	100	0.16 × 10^5^ ± 455	94.2 *	

Quercetin-3-*O*-α-l-arabinopyranoside	0 (control)	2.35 × 10^5^ ± 4984	-	75.3
	1	2.43 × 10^5^ ± 5173	0	
	5	2.14 × 10^5^ ± 4355	8.9 *	
	50	1.36 × 10^5^ ± 2893	42.1 *	
	100	0.87 × 10^5^ ± 1852	63.0 *	

Quercetin-3-*O*-β-d-galactopyranoside	0 (control)	2.35 × 10^5^ ± 4984	-	80.6
	1	2.47 × 10^5^ ± 5173	0	
	5	2.18 × 10^5^ ± 4355	7.2	
	50	1.50 × 10^5^ ± 2893	36.2 *	
	100	0.95 × 10^5^ ± 1852	59.6 *	

Quercetin	0 (control)	2.48 × 10^5^ ± 5987	-	<1.0
	1	1.11 × 10^5^ ± 3211	55.2 *	
	5	0.18 × 10^5^ ± 1101	92.7 *	
	50	0.15 × 10^5^ ± 578	94.0 *	
	100	0.13 × 10^5^ ± 499	94.8 *	

a Integrated area of chemiluminescence curve: mean of triplicate readings ± SD (*n* = 3);

b compared to the control;

* statistically significant difference (*p* < 0.05).

**Table 2:** Effect of phenolic compounds isolated from the methanolic extract of *Byrsonima crassa* on growth of *Helicobacter pylori* strain ATCC 43504. Results were expressed as means ± SD for three independent determinations.

Phenolic compound	Inhibition of bacterial growth (%)				

	Concentration (μg/mL)				

	64	128	256	512	1024
Amentoflavone	1.5 ± 0.3	4.3 ± 0.4	15.5 ± 0.6	15.9 ± 0.4	43.5 ± 0.6
(+)-Catechin	1.5 ± 0.2	8.8 ± 0.6	8.8 ± 0.4	10.3 ± 0.4	10.4 ± 0.5
Methyl gallate	5.8 ± 0.2	5.8 ± 0.4	7.3 ± 0.5	7.3 ± 0.3	7.4 ± 0.8
Quercetin-3-*O*-α-l-arabinopyranoside	8.2 ± 0.3	16.4 ± 0.4	17.4 ± 0.3	17.4 ± 0.6	17.8 ± 0.4
Quercetin-3-*O*-β-d-galactopyranoside	3.0 ± 0.6	3.5 ± 0.5	6.0 ± 0.6	9.7 ± 0.7	9.8 ± 0.7
Quercetin	7.9 ± 0.6	15.0 ± 0.8	17.4 ± 0.5	21.6 ± 0.8	47.4 ± 0.5

## References

[1] Mégraud F., Lehours P. (2007). *Helicobacter pylori* detection and antimicrobial susceptibility testing. Clin. Microbiol. Rev.

[2] Fischbach W., Malfertheiner P., Hoffmann J.C., Bolten W., Kist M., Koletzko S. (2009). *Helicobacter pylori* and gastroduodenal ulcer disease. Dtsch. Arztebl. Int.

[3] Konturek P.C., Konturek S.J., Brzozowski T. (2009). *Helicobacter pylori* infection in gastric cancerogenesis. J. Physiol. Pharmacol.

[4] Lee W.L., Harrison R.E., Grinstein S. (2003). Phagocytosis by neutrophils. Microbes Infect.

[5] Robinson J.M. (2008). Reactive oxygen species in phagocytic leukocytes. Histochem. Cell Biol.

[6] Bergamaschi A., Magrini A., Pietroiusti A. (2007). Recent advances in the treatment of *Helicobacter pylori* infection. Recent Pat. Antiinfect. Drug Discov.

[7] Kaboli S.A., Zojaji H., Mirsattari D., Talaie R., Derakhshan F., Zali M.R., Sheikhvatan M. (2009). Effect of addition of vitamin C to clarithromycin-amoxicillin-omeprazol triple regimen on *Helicobacter pylori* eradication. Acta Gastroenterol. Belg.

[8] Kamiji M.M., de Oliveira R.B. (2005). Non-antibiotic therapies for *Helicobacter pylori* infection. Eur. J. Gastroenterol. Hepatol.

[9] Sannomiya M., Fonseca V.B., da Silva M.A., Rocha L.R.M., dos Santos L.C., Hiruma-Lima C.A., Souza-Brito A.R.M., Vilegas W. (2005). Flavonoids and antiulcerogenic activity from *Byrsonima crassa* leaves extracts. J. Ethnopharmacol.

[10] Hirayama O., Takagi M., Hukumoto K., Katoh S. (1997). Evaluation of antioxidant activity by chemiluminescence. Anal. Biochem.

[11] Cai Y., Luo Q., Sun M., Corke H. (2004). Antioxidant activity and phenolic compounds of 112 traditional Chinese medicinal plants associated with anticancer. Life Sci.

[12] Heim K.E., Tagliaferro A.R., Bobilya D.J. (2002). Flavonoids antioxidants: Chemistry, metabolism and structure-activity relationships. J. Nutr. Biochem.

[13] Hsieha T., Liub T., Chiac Y., Chernd C., Lue F., Chuangd M., Mau S.Y., Chen S.H., Syu Y.H., Chen C.H. (2004). Protective effect of methyl gallate from *Toona sinensis* (Meliaceae) against hydrogen peroxide-induced oxidative stress and DNA damage in MDCK cells. Food Chem. Toxicol.

[14] Martini S., D’Addario C., Colacevich A., Focardi S., Borghini F., Santucci A., Figura N., Rossi C. (2009). Antimicrobial activity against *Helicobacter pylori* strains and antioxidant properties of blackberry leaves (*Rubus ulmifolius*) and isolated compounds. Int. J. Antimicrob. Agents.

[15] Bonacorsi C., Raddi M.S., Carlos I.Z., Sannomiya M., Vilegas W. (2009). Anti-*Helicobacter pylori* activity and immunostimulatory effect of extracts from *Byrsonima crassa* Nied. (Malpighiaceae). BMC Complement. Altern. Med.

[16] Funatogawa K., Hayashi S., Shimomura H., Yoshida T., Hatano T., Ito H., Hirai Y. (2004). Antibacterial activity of hydrolyzable tannins derived from medicinal plants against *Helicobacter pylori*. Microbiol. Immunol.

[17] Ustun O., Ozçelik B., Akyon Y., Abbasoglu U., Yesilada E. (2006). Flavonoids with anti-*Helicobacter pylori* activity from *Cistus laurifolius* leaves. J. Ethnopharmacol.

[18] Shin J.E., Kim J.M., Bae E.A., Hyun Y.J., Kim D.H. (2005). *In vitro* inhibitory effect of flavonoids on growth, infection and vacuolation of *Helicobacter pylori*. Planta Med.

[19] Arima H., Ashida H., Danno G. (2002). Rutin-enhanced antibacterial activities of flavonoids against *Bacillus cereus* and *Salmonella enteritidis*. Biosci. Biotechnol. Biochem.

[20] Vattem D.A., Lin Y.T., Ghaedian R., Shetty K. (2005). Cranberry synergies for dietary management of *Helicobacter pylori* infections. Process Biochem.

[21] Sannomiya M., Rodrigues C.M., Coelho R.G., dos Santos L.C., Hiruma-Lima C.A., Souza-Brito A.R.M., Vilegas W. (2004). Application of preparative high-speed counter-current chromatography for the separation of flavonoids from the leaves of *Byrsonima crassa* Niedenzu (IK). J. Chromatogr.

[22] Galice D.M., Bonacorsi C., Soares V.C., Raddi M.S., Fonseca L.M. (2006). Effect of subinhibitory concentration of chlorhexidine on *Streptococcus agalactiae* virulence factor expression. Int. J. Antimicrob. Agents.

